# Niche and Neutral Processes Together Determine Diversity Loss in Response to Fertilization in an Alpine Meadow Community

**DOI:** 10.1371/journal.pone.0134560

**Published:** 2015-08-17

**Authors:** Wei Li, Ji-Min Cheng, Kai-Liang Yu, Howard E. Epstein, Guo-Zhen Du

**Affiliations:** 1 State Key Laboratory of Soil Erosion and Dryland Farming on the Loess Plateau, Northwest A&F University, Yangling, 712100, Shaanxi, P. R. China; 2 Institute of Soil and Water Conservation of Chinese Academy of Sciences & Ministry of Water Resource, Yangling, 712100, Shaanxi, P. R. China; 3 Department of Environmental Sciences, University of Virginia, Charlottesville, VA, 22904–4123, United States of America; 4 School of Life Sciences, Lanzhou University, Lanzhou, 730000, P. R. China; North Carolina State University, UNITED STATES

## Abstract

Fertilization via nutrient deposition and agricultural inputs is one of the most important factors driving decreases in plant diversity. However, we still do not fully understand which processes (niche process or neutral process) are more important in leading to decreases in plant diversity caused by fertilization. A hypothesis-based approach was used to test the relative importance of niche versus neutral processes along a fertilization gradient in an alpine meadow community on the eastern Tibetan plateau, China. Niche overlap values were calculated for species biomass, and the null model was used to generate the values of niche overlap expected at random. A linear regression modeling was used to evaluate the relationship between functional traits (specific leaf area, leaf dry matter content, and leaf total nitrogen concentration) and species relative abundance. Our results demonstrated that observed niche overlap for species biomass was significantly higher than expected at lower fertilization gradients. Moreover, we also found a significantly negative correlation between species relative abundance and specific leaf area and leaf dry matter content, but a significantly positive correlation between relative abundance and leaf nitrogen concentration at lower fertilization gradients. However, these relationships were not significant at higher fertilization gradients. We concluded that community assembly is dynamic progression along the environmental gradients, and niche and neutral processes may together determine species diversity loss in response to fertilization.

## Introduction

A large number of experiments have shown that fertilization leads to a decrease in species diversity and a shift in community composition [1, 2]. Although these negative effects of fertilization on species diversity are well documented by using experimental assemblages [3], the processes responsible for these changes in natural communities are not well understood [4]. Community assembly is regarded as a dynamic progression that is driven by processes such as dispersal, the responses of species to environment, and the biotic interactions (i.e., competition and predation) [5]. More recently, ecologists are now revisiting these processes in order to understand and predict the effects of environmental changes, such as fertilization, on communities. Therefore, a key aim of this work is to evaluate the relative importance of different processes on biological communities.

Two distinct views which have been often proposed to explain community structuring are niche assembly and dispersal assembly [6]. In general, niche processes associated with competition and environmental filtering [7] and neutral processes associated with random demographic dynamics [6] determine community composition and structure. In addition, multiple processes can act simultaneously [7]. For example, two opposing niche-based processes (environmental filtering and competitive exclusion) can drive community assembly. Environmental filtering proposes that species occurring together in communities and thus experiencing the same environmental conditions are likely to share similar functional traits [8]. Competitive exclusion proposes that competition is likely to limit the similarity of species functional traits in these environments [9]. Neutral processes predicate that all species are identical in their demographic rates (birth, death, dispersal and speciation rates) and exclusion processes are completely random [6]. Predictions from neutral theory are contrary to the assumptions of niche theory, which asserts that species-specific traits and trade-offs determine the coexistence of species and maintenance of diversity within a community [10, 11]. However, it is difficult for niche theory to explain very high species diversity, such as in tropical rain forests [12]. Actually, niche theory and neutral theory alone cannot explain many patterns observed in nature. Researchers have attempted to incorporate both of them into more general models [13–17]. Gravel et al. [16] assumes that communities are located in a continuum (niche and neutrality form ends of a continuum from niche-structured communities to neutral structure). According to their predictions, diversity and abundance patterns along environmental gradients will be the consequence of the balance between stochastic processes and competitive exclusion.

The Tibetan Plateau is the youngest and highest plateau in the world. Alpine meadows comprise the representative vegetation on the plateau, and they are also very fragile and sensitive ecosystems due to changes in global climate and land use [18]. Previously, a series of fertilization experiments were conducted in an alpine meadow on the Tibetan Plateau to better understand the potential mechanism of species loss due to fertilization [19–21]. To our knowledge, no previous study has empirically evaluated the relative importance of niche and neutral processes as determinants of community structure and diversity along a fertilization gradient. Furthermore, a definitive answer to what extent communities are structured by niche or neutral processes can be obtained by combining theoretical models with manipulative experiments [22]. Here, we test two hypotheses that derive from neutral and niche theories to evaluate the relative importance of these two different processes along a fertilization gradient.

We firstly focus on the niche overlap between pairs of species. If competitive exclusion is the primary factor in determining community composition, observed overlap between pairs of species will be lower than expected at random. If environmental filtering effects predominate, observed overlap will be higher than expected at random. The lack of a significant difference from expected at random may indicate either a balance between the two mechanisms (competitive exclusion and environmental filtering) or that neutral processes control community assembly [23]. Therefore, we can test the assembly processes by comparing actual niche overlap in the fields with that expected at random in a null model along the environmental gradients [24].

Second, neutral theory predicts that all individuals are assumed to be functionally equivalent [6]. Therefore, there is no relationship between species functional traits and their relative abundance. In contrast, niche theory suggests that a mechanistic model based on functional traits could predict patterns of species relative abundance. We investigated the relationship between three leaf functional traits and species relative abundance: (1) specific leaf area (SLA, the ratio of water-saturated leaf area to leaf dry mass; (2) leaf total nitrogen concentration (LNC) and (3) leaf dry matter content (LDMC, the ratio of leaf dry mass to water-saturated fresh mass). SLA is an important variable in comparative plant ecology because it is closely related with relative growth rate [25] and leaf net assimilation rate [26]; it is also a good predictor of plant response to resource availability [27]. LNC is closely correlated with protein concentrations involved in photosynthesis as well as leaf growth and defense strategies [28]. LDMC is tied to plant nutrient retention and water [29]. Fast growing species from nutrient-rich habitats usually have high SLA, high LNC and low LDMC, while opposite trends characterize species from nutrient-poor habitats [30]. These responses reflect a fundamental trade-off (leaf economics spectrum) between traits related to nutrient conservation and traits related to nutrient acquisition and turnover [31].

Based on the above two hypotheses, the main objectives of this study were to: (a) determine empirically the relative importance of niche and neutral processes along a fertilization gradient; and (b) evaluate whether soil resource availability alters the relationship between leaf functional traits and species relative abundance in this alpine meadow.

## Material and Methods

### Study site

**Fig 1:**
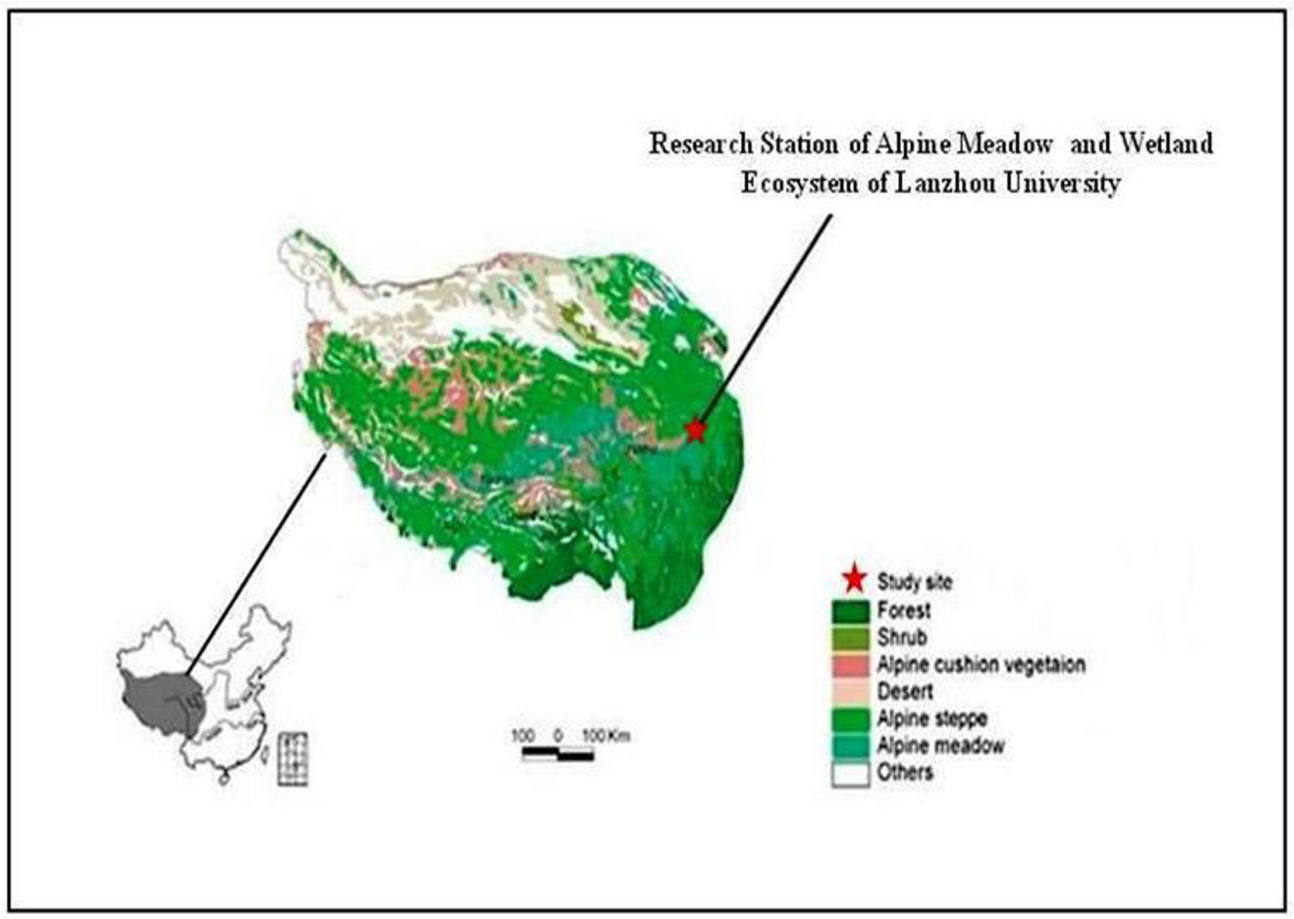
The location of the Tibetan Plateau in China and the Research Station of Alpine Meadow and Wetland Ecosystems of Lanzhou University at the Tibetan Plateau. The land cover of the Tibetan Plateau is based on the vegetation map of China (Hou et al. 1982).

The experiment was conducted at the Research Station of Alpine Meadow and Wetland Ecosystems of Lanzhou University (N 33°58′, E101°53′) on the eastern Tibetan plateau, 3500 m a.s.l., China (Fig 1). The average yearly temperature is 1.2°C, ranging from -10°C in January to 11.7°C in July, with about 270 frost days. Average annual precipitation over the last 35 years is 620 mm, occurring mainly during the short, cool summer. The annual cloud-free solar radiation is about 2580 h [21]. The subalpine meadow soil has relatively low P availability (< 2mg available P kg^−1^ dry soil). The vegetation, typical of Tibetan alpine meadows, is dominated by clonal *Kobresia* spp., *Elymus nutans*, *Festuca ovina*, *Poa poophagorum*, *Agrostis* spp., *Saussurea* spp. and *Anemone rivularis* [21,32]. The experimental site has been overgrazed in the past, but has been fenced and only grazed in winter and early spring (October to April in the following year) since 2006.

### Experimental design

Thirty-six 4 × 10-m^2^ plots composed of four fertilization levels with nine replicates were distributed in nine columns and four rows with a randomized block design. Each plot was separated from the others by a 2-m buffer strip. The fertilization treatments were generated with different amounts of (NH_4_)_2_ HPO_4_ fertilizer applied annually at the beginning of the growing season (usually in the middle of May) from 2007 to 2010. The fertilizer was applied during a moderate rain event to avoid artificial watering [21]. Fertilizer applications of 0, 15, 30 and 60 g m ^-2^ yr ^-1^ are hereafter referred to as F0, F15, F30 and F60, which corresponds to 0, 3.15, 6.3, and 12.6 g N m^-2^ yr ^-1^ and 0, 3.5, 7.0 and 14.0 g P m^-2^ yr^-1^, respectively. Each plot was separated into two subplots: a 4 × 4 m subplot for vegetation monitoring, and a 4 × 6 m subplot for individual plant sampling.

### Community measurements

Community measurements were conducted from 2 to 4 Sept 2010 when biomass reaches its peak. One 0.25 m^2^ quadrat was harvested from the 4 × 4 m subplot in each plot. The quadrat location was randomly selected with the constraint that it was at least 0.5 m from the margin to avoid edge effects. We estimated the cover of each species in each quadrat before being clipped and brought to the lab. For clonal species, an individual plant was defined as a group of tillers connected by a crown [19]. The cover of each species in each plot was estimated as a percentage using a canopy interception technique based on cardboard cut-outs of various shapes and sizes as visual guides. All samples were dried at 80°C for 48 h, and weighed to the nearest 0.01 g.

In each plot, we measured light with a Decagon Sunfleck ceptometer (Decagon, Pullman, Washington, DC) at the time of vegetation monitoring on 2 Sept 2011. Light readings were taken on a cloudless day (11:00–13:00h). Photosynthetically active radiation (PAR) was recorded at 10cm above the soil surface and at the top of the canopy. The ratio between these two measurements was taken as a proxy for PAR reaching the understory.

### Leaf trait measurements

Following Cornelissen et al. [33], we measured three functional traits (SLA, LNC and LDMC) on the 25 most common species in each fertilization gradient in early September 2010. These species represented 85–95% of the peak standing biomass and 80–90% of the vegetation cover of the total plant community in the studied plots.

We randomly sampled 2 individuals and 6 mature leaves (3 leaves per individual) at flowering time for each of the 25 species in each 4 × 6 m subplot. That is, 18 individuals and 54 mature leaves were measured for each of the 25 species in each fertilization level. Leaves were scanned to measure leaf area in the field, and fresh weight of leaves was determined with a balance (Acculab Lt-320; Acculab, Measurement Standards Inc., Danvers, MA, USA). Following these measurements, leaves were placed in paper bags and dried in the sun. Leaf samples were oven-dried at 80°C for 48 h in the laboratory and their dry masses were measured on a semianalytical balance with an accuracy of 10^−4^ g (Sartorius AG, Goettingen, Germany). Dried leaves samples were ground using a ball mill (NM200; Retsch, Haan, Germany). Total N concentrations of leaves were determined using an elemental analyzer (2400 II CHNS/O Elemental Analyzer; Perkin-Elmer, Boston, MA, USA).

## Data Analysis

From the vegetation harvest data, two indices were selected to estimate diversity according to Pielou [34]. The first index is plant species richness, represented by the number of species recorded in each quadrat. The second, Shannon–Weiner diversity index is: $H^{'}=\sum_{i=1}^{s}P_{i\;}\log_{2}\;P_{i}$, where pi is the cover proportion of species represented by species i.

We used one-way ANOVA to test the effect of fertilization on PAR in the understory, species diversity (species richness and Shannon-Wiener diversity index), aboveground community biomass and each leaf functional trait. Then, we performed a linear regression modeling to test the relationship between functional traits and species relative abundance. Independent manipulation of SLA, LDMC and LNC would be required to truly disentangle these effects. Although this approach is correlative in nature, it can provide a way to test the importance of these mechanisms (functional traits-based niche assembly and dispersal-based neutral assembly). All variables met the statistical assumptions (residuals normality, homogeneity of variance and data linearity) when tested using the Shapiro-Wilk test and Levene’s test, respectively. These analyses were performed with SPSS15.0.

For each pair of species, based on the species biomass, a mean overlap value of entire community is calculated along the niche axis according to a function [24]: $I_{\mathrm{Obs}}=\sum_{i=1}^{s}\sum_{j=i+1}I_{Nij\;}P_{i}P_{j}$, where I_Obs_ is observed species biomass overlap, S is species richness in the community, I_Nij_ is the distance between the positions of species i and j on the species biomass axis, and P_i_ and P_j_ represent proportional abundance of species i and j, respectively. The null model was used to generate the values of niche overlap expected at random. The species composition of all simulated communities was exactly the same as that observed, with only the distribution of relative abundances among species changing [24]. When species relative abundances are perfectly even, or when niche overlap is identical between all species pairs, the randomized niche overlap values of a sample will always be identical to that observed. The potential for the randomized overlap svalues to differ from the observed values tends to increase as species biomass become less even. These effects could be in either direction, so that they do not introduce any bias [24].

A total of 10^4^ randomizations were used in all analyses. P-values were calculated as the proportion of randomizations giving a distance value or more extreme than that observed, with the P-values being doubled to give a one-tailed test based on a 95% confidence interval test. F statistics were considered significant at α = 0.05 [24]. This process was made using the software R (R Development Core Team Version 2.10.1. 50720).

## Results

### Species diversity

**Fig 2:**
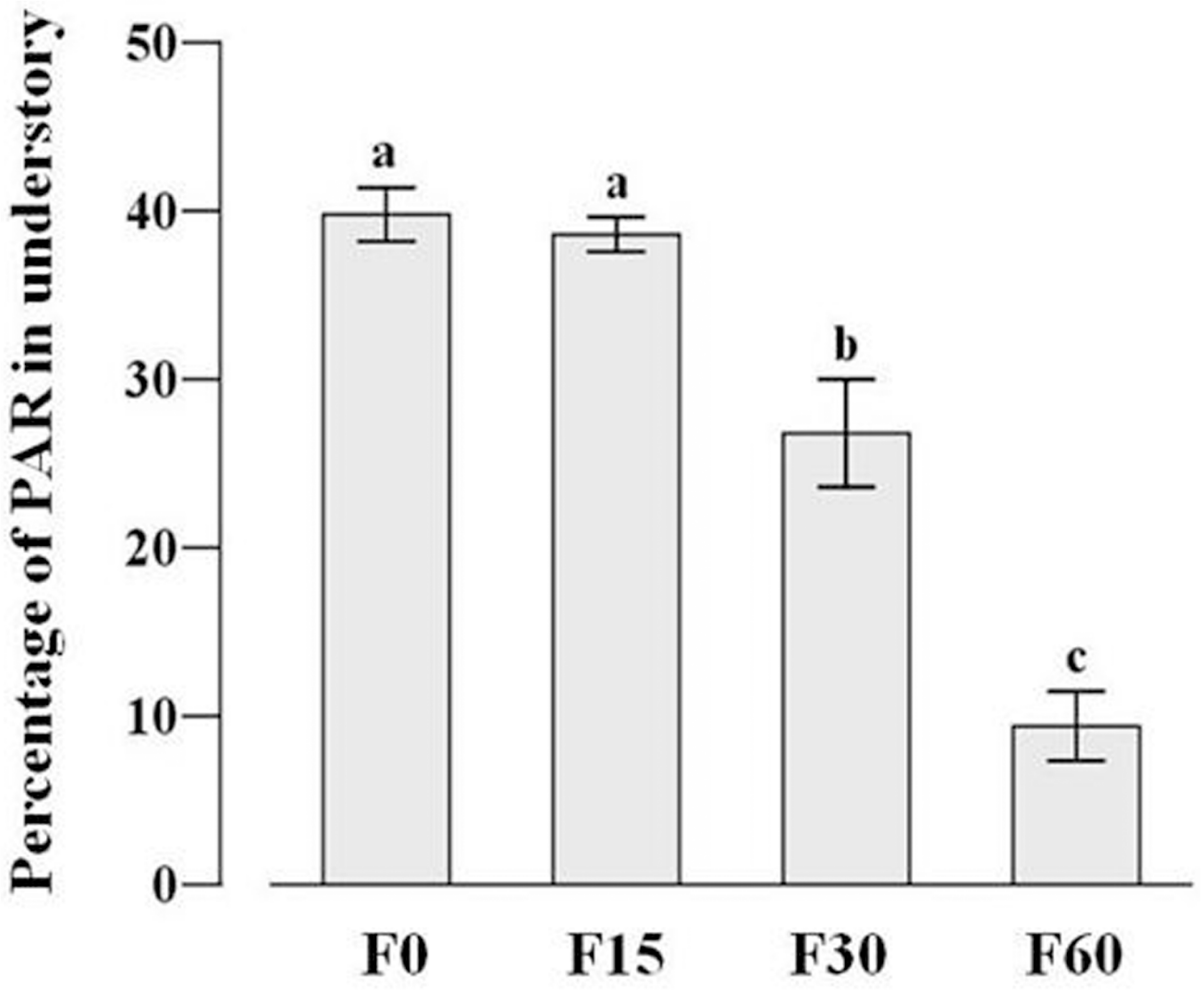
Effects of fertilization on PAR in the understory (~10cm near the soil surface). F0, F15, F30 and F60 represent (NH_4_)_2_HPO_4_ fertilizer applications of 0, 30, 60 and 120g m^−2^ year^−1^. PAR, Photosynthetically active radiation.

**Fig 3:**
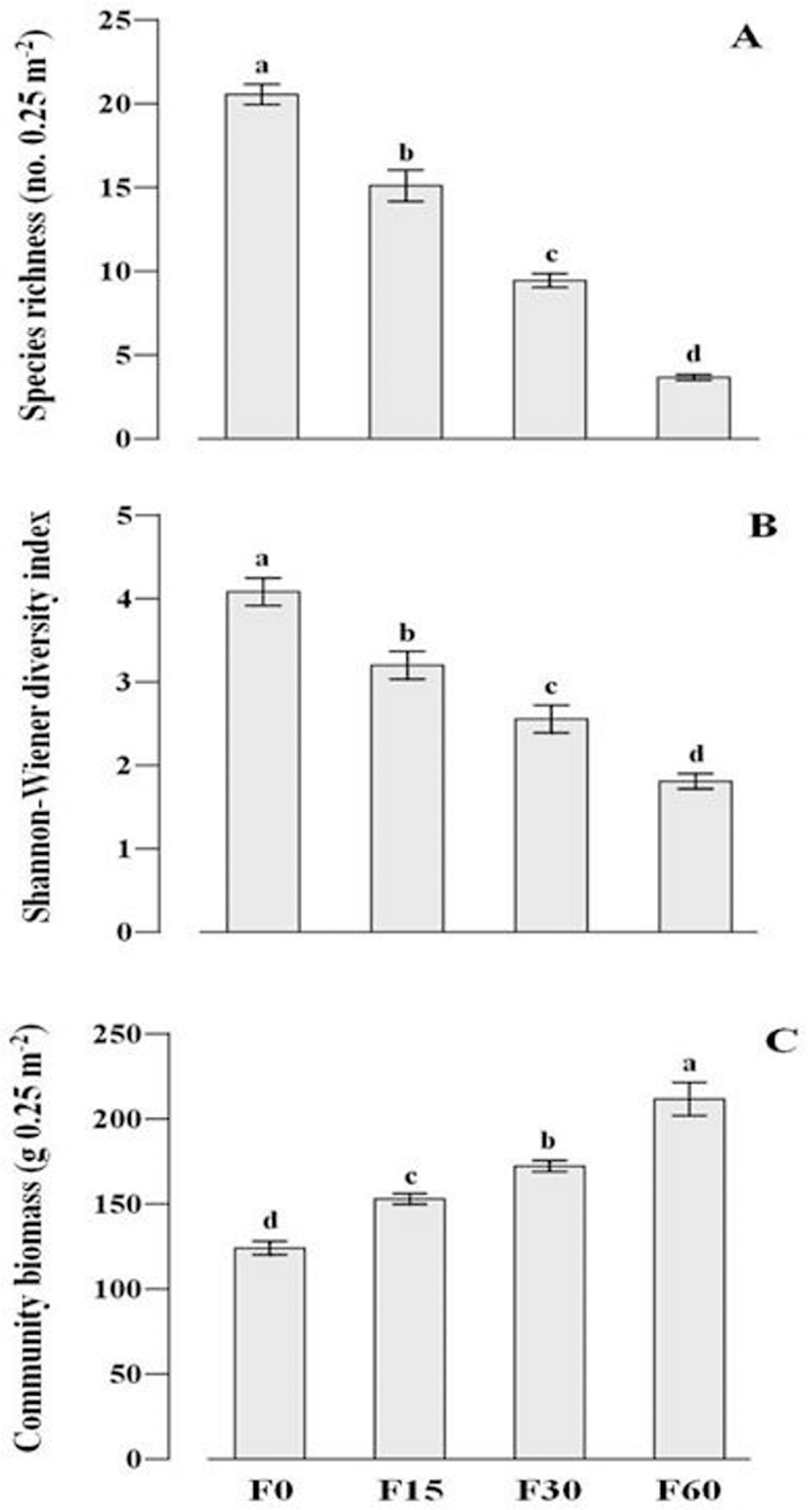
The changes of species richness (A), Shannon-Wiener diversity index (B)and community biomass (C) along the fertilization gradients. F0, F15, F30, and F60 represent (NH_4_)_2_HPO_4_ fertilizer applications of 0, 15, 30 and 60 g m^-2^ yr^-1^.

As expected, fertilization significantly decreased the PAR in the understory (*F*_*3*,*8*_ = 176.981, *P*<0.001) (Fig 2), and species diversity always decreases with increased artificial fertilization levels. Fertilization significantly reduced species richness (*F*_*3*,*8*_ = 147.445, *P*<0.001) (Fig 3A) and Shannon–Weiner diversity index (*F*_*3*,*8*_ = 40.534, *P*<0.001) (Fig 3B), but significantly increased aboveground community biomass (*F*_*3*,*8*_ = 41.125, *P*<0.001) (Fig 3C).

### Species functional traits and relative abundance

**Fig 4:**
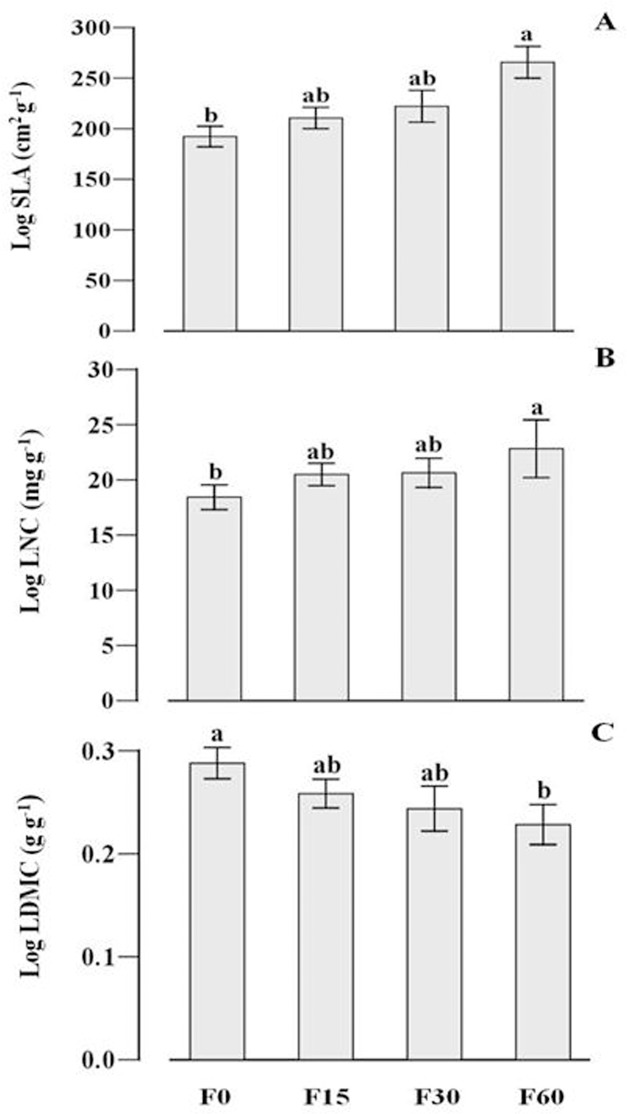
The changes of specific leaf area (SLA) (A), leaf nitrogen concentration (LNC) (B) and leaf dry matter content (LDMC) (C) along the fertilization gradients. Bars with different letters denote significant difference (P = 0.05); F0, F15, F30, and F60 represent (NH_4_)_2_HPO_4_ fertilizer applications of 0, 15, 30 and 60 g m^-2^ yr^-1^.

**Fig 5:**
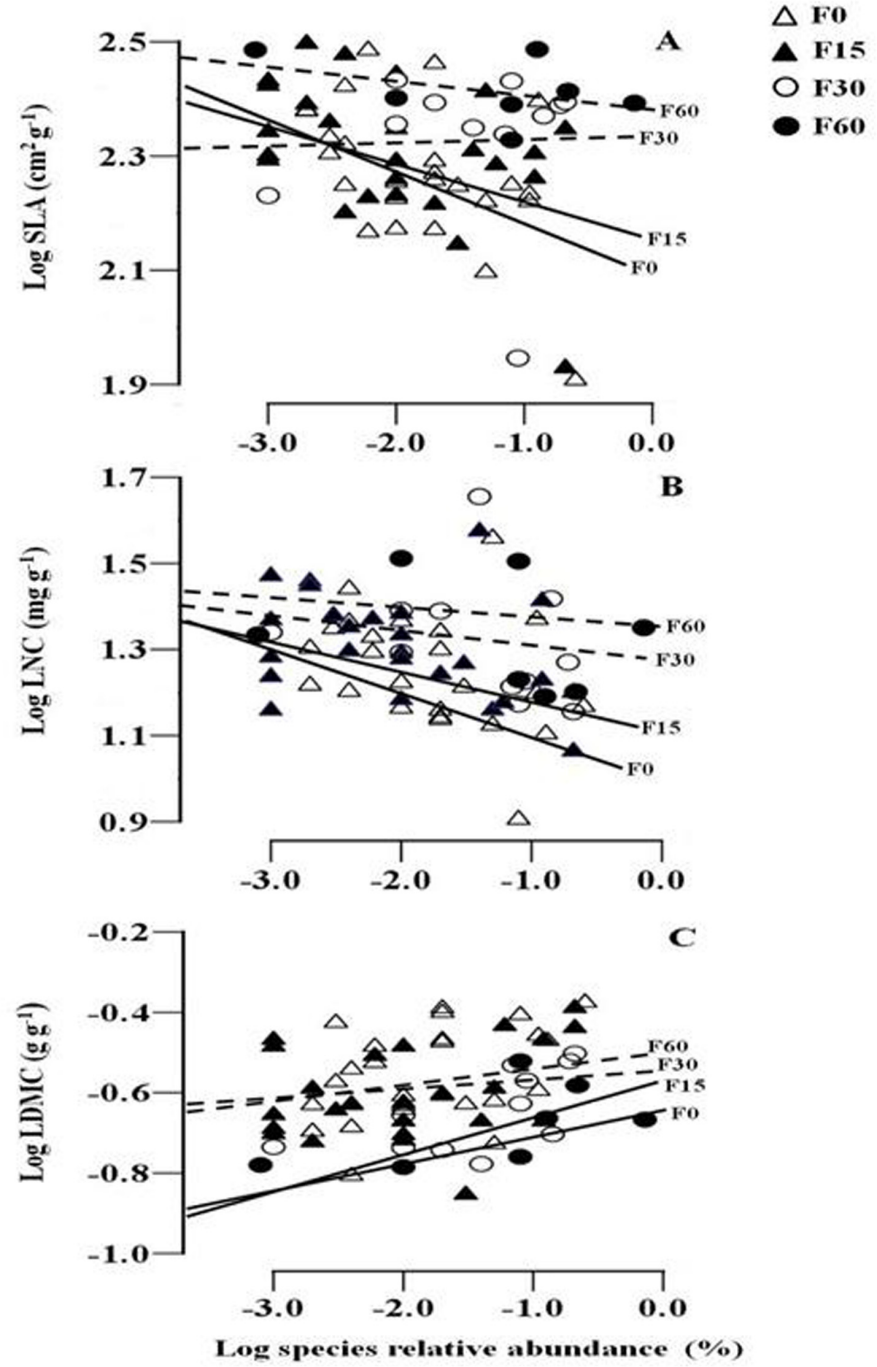
The relationship between species relative abundance and specific leaf area (SLA) (A), leaf nitrogen concentration (LNC) (B) and leaf dry matter content (LDMC) (C) along a fertilization gradient. Significant relationships (P < 0.05) are denoted with solid lines. F0, F15, F30, and F60 represent (NH_4_)_2_HPO_4_ fertilizer applications of 0, 15, 30 and 60 g m^-2^ yr^-1^.

Fertilization had a significant influence on SLA (*F*_*3*,*63*_ = 2.089, *P* = 0.011) and LNC (*F*_*3*,*63*_ = 3.945, *P* = 0.023) and LDMC (*F*_*3*,*63*_ = 2.843,*P* = 0.038) (Fig 4A, 4B and 4C;Table 1). We found a significantly negative correlation between species relative abundance and SLA (Fig 5A; Table 2) and LNC (Fig 5B; Table 2), but a significantly positive correlation between relative abundance and LDMC (Fig 5C; Table 2) at lower fertilization gradients (F0 and F15). For the higher fertilization gradients, the relationships were not significant (F30 and F60).

**Table 1:** The effect of fertilization on SLA, LNC and LDMC of component species.

	SLA				LNC				LDMC			
	F0	F15	F30	F60	F0	F15	F30	F60	F0	F15	F30	F60
*Elymus nutans*	166.008	182.349	244.780	247.113	15.767	16.900	18.633	22.409	0.348	0.339	0.301	0.271
*Kobresia capillifolia*	80.825	84.837	88.354		14.698	15.833	16.833		0.421	0.408	0.378	
*Anemone rivularis*	171.140	201.268	234.654	245.737	23.433	25.867	26.167	31.987	0.254	0.213	0.198	0.174
*Sphallerocarpus gracilis*	179.541	222.640	247.617	252.232	16.711	19.568	24.500	32.479	0.228	0.213	0.181	0.164
*Anemone obtusiloba*	185.847	195.484	227.149		19.897	21.533	24.600		0.247	0.237	0.183	
*Anemone trullifolia*	177.137	219.909			15.933	19.133			0.206	0.198		
*Agrostis hugoniana Rendle*	248.568	258.347	269.966	306.820	12.697	14.400	14.900	14.812	0.338	0.257	0.236	0.217
*Aster alpinus*	148.630	170.103			14.568	15.233			0.233	0.191		
*Euphorbia micractina*	289.840	277.698	270.987	335.753	14.400	18.933	19.634	219.569	0.335	0.327	0.221	0.209
*Gentianopsis paludosa*	264.227	314.104			23.100	28.700			0.156	0.189		
*Halenia elliptica*	239.142	265.147			20.067	23.333			0.201	0.221		
*Delphinium kamaonense*	168.088	181.651			23.167	24.157			0.246	0.197		
*Saussurea stella*	124.798	139.584			13.300	18.467			0.187	0.140		
*Thalictrum alpinum*	215.092	245.806			23.900	28.033			0.375	0.257		
*Ranunculus tanguticus*	201.369	228.647			22.300	23.867			0.267	0.227		
*Leontopodium nanum*	305.642	299.973			21.300	22.467			0.298	0.235		
*Festuca ovina*	195.342	199.564			13.700	14.400			0.407	0.327		
*Trollius farreri*	146.879	158.567	170.160		19.633	19.767	21.867		0.327	0.234	0.184	
*Poa poophagorum*	177.569	222.318	248.224	259.150	8.033	11.567	14.300	16.987	0.392	0.364	0.337	0.262
*Potentilla fragarioides*	148.255	168.612			21.933	23.433			0.340	0.310		
*Koeleria cristata*	180.861	192.504	217.987	222.896	13.867	14.978	16.367	15.547	0.397	0.368	0.294	0.301
*Plantago depressa*	239.746	269.912			16.467	17.233			0.234	0.204		
*Saussurea nigrescens*	176.654	164.143			16.300	17.469			0.235	0.249		
*Geranium pylzowianum*	208.691	196.163			27.567	29.567			0.287	0.340		
*Thermopsis lanceolata*	165.909	203.569	223.678		36.167	37.533	45.200		0.239	0.213	0.167	

Notes: SLA, leaf area per unit dry mass; LDMC, leaf dry matter content; LNC, leaf total nitrogen concentration. F0, F15, F30 and F60 represent (NH_4_)_2_HPO_4_ fertilizer applications of 0, 15, 30 and 60 g m^-2^ yr^-1^.

**Table 2:** Linear regression models fitted to the relationship between species relative abundance and SLA, LNC and LDMC along the fertilization gradient evaluated.

Treatments	n	SLA		LNC		LDMC	
		R^2^	P	R^2^	P	R^2^	P
F0	25	0.256	**0.013**	0.196	**0.035**	0.176	**0.032**
F15	25	0.216	**0.019**	0.173	**0.041**	0.148	**0.044**
F30	11	0.005	0.836	0.175	0.594	0.145	0.370
F60	7	0.205	0.298	0.476	0.317	0.263	0.242

Notes: n refers to the number of species in each fertilization gradient. P <0.05 are in bold. SLA, leaf area per unit dry mass; LDMC, leaf dry matter content; LNC, leaf total nitrogen concentration. F0, F15, F30 and F60 represent (NH_4_)_2_HPO_4_ fertilizer applications of 0, 15, 30 and 60 g m^-2^ yr^-1^.

### Biomass overlaps of pairs of species for entire community

The biomass overlap was significantly higher than expected at random at lower fertilization gradients (F0 and F15) (Table 3), which provides the evidence of environmental filtering (Table 3). Observed biomass overlap between pairs of species demonstrated a stochastic distribution at the higher fertilization gradients (F30 and F60), which indicates the occurrence of neutral processes (Table 3).

**Table 3:** Observed biomass overlap (I_Obs_; with confidence intervals (CIs) in terms of null models) in different fertilization gradients.

Treatments	I_obs_	95%CI
F0	**0.0454**	0.0156–0.0349
F15	**0.0398**	0.0118–0.0246
F30	0.0614ns	0.0431–0.0845
F60	0.4071ns	0.1374–0.5927

Notes: Statistical tests were performed against the null expectation that species abundances within each fertilization gradient were unrelated to leaf functional traits; all tests were one-tailed; Bold types indicate a significant difference based upon 95% CI; ns, Not significant; F0, F15, F30 and F60 represent (NH_4_)_2_HPO_4_ fertilizer applications of 0, 15, 30 and 60 g m^-2^ yr^-1^.

## Discussion

Plant ecologists have long concentrated on explaining the decline of species diversity along artificial fertilization gradients [1, 2, 10]. Consistent with these studies, our results demonstrated that species richness and Shannon-Wiener diversity index significantly decreased with increased community biomass along a fertilization gradient. We have used a hypothesis-based approach to test the relative importance of niche versus neutral processes in explaining species diversity loss after fertilization. Our results showed that niche and neutral processes may together determine species loss due to fertilization in an alpine meadow community (Table 3, Fig 5).

The question of whether functional traits can determine species relative abundance has intrigued ecologists for several decades, and the results have varied substantially [35, 36]. Shipley [37] recently proposed a ‘community assembly through trait selection’ (CATS) model. This model predicts that species relative abundances are constrained by their functional traits, but with a strong stochastic component that recognizes the importance of chance events affecting community composition. Cornwell and Ackerly [38] found that functional trait–species abundance relationships mainly depend on the measured scale. No significant relationships were found at a landscape scale; the significant relationships appeared at a local scale. Reader [39] found that species relative abundances correlated with plant growth rate, shoot mass, root to shoot radio, and leaf palatability in infertile ridge top habitats; but Lloret et al. [40] found that plant life history traits appeared to play an unimportant role in influencing species abundance in Mediterranean islands. In this study, we found significant relationships between species abundance and three functional traits at lower fertilization gradient. In contrast, we found no significant relationship between traits and abundance at higher fertilization gradients. Our findings were partially consistent with the model prediction [36, 41] and field manipulations experiments [42]. Our results demonstrated that the functional traits-species abundance relationships are dependent on environmental context (soil nutrient and light). At low or moderate soil nutrient, species traits and trade-offs in community could be the determinant of species abundance or dominance. However, there will be no correlation between abundance and traits at high soil nutrient due to a series of stochastic events. For example, species must be tolerant of low light to survive under the canopy. In addition, the pattern of traits and abundance will depend on the spatial and temporal frequency distribution of microenvironments as well as the connection between traits and microenvironments [43].

Plant functional traits can reflect evolutionarily derived strategies of resource capture and species interactions, which influence community composition and structure [44, 45]. In this study, community composition has a shift from lower fertilization gradient to higher fertilization gradient. Four years after the start of the experiment, species with higher SLA, LNC and lower LDMC have occurred together, which reflected well-characterized ecological strategies (namely to maximize resource acquisition) under low light and high nutrient environments [46]. Species that cannot tolerate such environment pressure (shading and thus light competition) would be excluded. Meanwhile the biotic interactions gradually shift from below-ground competition dominated when soil resources are limited to above-ground competition dominated when soil resources are abundant but shading is intense along the fertilization gradients [3, 47]. The transition of competition increases above-ground productivity while decreasing the availability of light in the understory (Fig 2), which leads to greater mortality or competition exclusion of small-sized species [48, 49]. In our study, the graminodis (e.g. *Elymus nutans* and *Poa poophagorum*) that were predicted to be better light competitors were abundant after fertilization due to their tall stature and high relative growth rates [43, 50–52]. Our results are congruent with previous studies showing that the nutrients conservation graminoids were priority selection after fertilization [53], because graminoids generally had thinner, denser leaves than forbs [54]. In addition, a few forbs (e.g. *Anemone rivularis* and *Sphallerocarpus gracilis*) ultimately survived in the understory because of their higher SLA and LNC. Harpole and Tilman [42] also found the evidence that plant functional trait values could control species abundance along a nitrogen gradient, which is consistent with niche theory. Our recent study [55] also found that non-neutral, trait-based processes play an important role in determining species abundance in different fertilization habitats. In particular, species that most effectively capture light (tall or higher SLA) frequently outcompete species that are less effective at light capture (forbs and species in the lower canopy or with lower SLA) [56]. In contrast, neutral models predicted that species are essentially equivalent in their demographic rates, independently of their functional traits [57].

Some studies have demonstrated that niche process can cause traits convergence related to habitat selection and traits divergence related to resource competition [23]. These results were partially consistent with our findings. In this study, niche overlap was significantly higher than expected at random at lower fertilization gradients, thus providing the evidence of environmental filtering. As a consequence of environmental filtering, a significant relationship between species traits and abundance is emerged [58]. Environmental filtering can force species to converge toward an optimum trait value and become functionally similar. After exclusion of grazing or fertilization, this meadow tends to be dominated by tall, fast-growing species (higher SLA, LNC and lower LDMC) that can develop a disproportionately large competitive effect on local resources [43]. Some recent studies also report that fertilization can increase similarity in species composition between communities [59]. In contrast, observed species niche overlap showed no significant difference with our randomized expectations at higher fertilization gradients thus emphasizing the evidence of neutral processes. This may arise from the balance between competitive exclusion and environmental filtering. Species with different combination of traits will ultimately emerge separation at higher fertilization gradients. Taller species (e.g. *E*. *nutans* and *P*.*poophagorum*) occupy the canopy layer, yet the lower, larger SLA species (e.g. *A*. *rivularis* and *S*. *gracilis*) dominated in the understory. In our previous study [60], we have found that the trait-based deterministic process and dispersal-based stochastic process may be equally important in structuring plant community in different habitats. At the same time, this result also confirms that species with different traits can co-exist because of contrasted shade tolerances and N utilization strategies [61].

## Conclusion

By evaluating the niche overlaps and the relationship between species abundance and functional traits along a fertilization gradient, we found that fertilization significantly increased the overlaps of biomass at lower fertilization gradients. However, with increased fertilization gradient, there were no significant correlations between species functional traits and relative abundance. We concluded that niche and neutral processes may together determine species diversity loss in response to fertilization.
